# The chicken erythrocyte epigenome

**DOI:** 10.1186/s13072-016-0068-2

**Published:** 2016-05-24

**Authors:** Sanzida Jahan, Wayne Xu, Shihua He, Carolina Gonzalez, Geneviève P. Delcuve, James R. Davie

**Affiliations:** Children’s Hospital Research Institute of Manitoba, University of Manitoba, 715 McDermot Avenue, Room 600A, Winnipeg, MB R3E 3P4 Canada

**Keywords:** Chromatin fractionation, Chromosomal domains, Histone acetylation, H3K27ac, Histone methylation, H3K4me3, Chicken erythrocyte transcriptome

## Abstract

**Background:**

Transcriptional regulation is impacted by multiple layers of genome organization. A general feature of transcriptionally active chromatin is sensitivity to DNase I and association with acetylated histones. However, very few of these active DNase I-sensitive domains, such as the chicken erythrocyte β-globin domain, have been identified and characterized. In chicken polychromatic erythrocytes, dynamically acetylated histones associated with DNase I-sensitive, transcriptionally active chromatin prevent histone H1/H5-induced insolubility at physiological ionic strength.

**Results:**

Here, we identified and mapped out all the transcriptionally active chromosomal domains in the chicken polychromatic erythrocyte genome by combining a powerful chromatin fractionation method with next-generation DNA and RNA sequencing. Two classes of transcribed chromatin organizations were identified on the basis of the extent of solubility at physiological ionic strength. Highly transcribed genes were present in multigenic salt-soluble chromatin domains ranging in length from 30 to over 150 kb. We identified over 100 highly expressed genes that were organized in broad dynamically highly acetylated, salt-soluble chromatin domains. Highly expressed genes were associated with H3K4me3 and H3K27ac and produced discernible antisense transcripts. The moderately- and low-expressing genes had highly acetylated, salt-soluble chromatin regions confined to the 5′ end of the gene.

**Conclusions:**

Our data provide a genome-wide profile of chromatin signatures in relation to expression levels in chicken polychromatic erythrocytes.

**Electronic supplementary material:**

The online version of this article (doi:10.1186/s13072-016-0068-2) contains supplementary material, which is available to authorized users.

## Background

Histone acetylation plays a critical role in the structure of transcriptionally active chromatin. The seminal studies of Weintraub and Groudine demonstrated that transcribed chromatin has an increased sensitivity to DNase I (approximately twofold to threefold greater than the bulk of chromatin) [1]. The dynamically acetylated histones bound to transcribed chromatin are largely responsible for this DNase I sensitivity. Genomic mapping of acetylated histones (H3K9/14ac, H4K16ac) demonstrated that the acetylated histones are located around the transcription start site of expressed genes [2–4]. However, for α- and β-globin genes in mammalian and chicken erythroid cells, the dynamically highly acetylated histones are broadly distributed to encompass transcriptionally competent and active globin genes. These extensive acetylation patterns display sharp edges where acetylation drops abruptly, defining acetylation domains [5–7]. The boundaries of the acetylated β-globin domain co-map with those of the DNase I-sensitive β-globin chromatin domain [8]. The dynamically acetylated histones also render the active/competent chromatin soluble at low ionic strength (50–150 mM NaCl), by preventing histone H1/H5-mediated chromatin insolubility at physiological ionic strength [9, 10]. In parallel with the decline in acetylated histones and DNase I sensitivity, the chromatin salt solubility at physiological ionic strength falls sharply at the 5′ boundary of the β-globin domain [11].

The DNase I sensitive and dynamically highly acetylated chromatin of the 33-kb chicken erythroid β-globin domain is one of the better characterized domains [12]. Other DNase 1-sensitive domains containing one or more expressed genes have been mapped in chicken and mammalian cells. In the chicken hen oviduct, the *SERPINB14* (ovalbumin) gene and two pseudogenes are in a 100-kb DNase I-sensitive domain [13], while the *GAPDH* gene lies in a 15-kb DNase I-sensitive domain [14]. In human hepatocytes, the *APOB* gene resides in a 50-kb DNase I-sensitive domain [15]. Within the DNase I-sensitive domains are regions of hypersensitivity (about 100-fold more sensitive than bulk chromatin), which are nucleosome-depleted regions associated with regulatory elements such as enhancers, locus control regions and promoters.

The study of the chromatin structure of chicken mature erythrocytes and polychromatic erythrocytes from anemic birds has advanced the field. Polychromatic erythrocytes are transcriptionally active, while mature erythrocytes are transcriptionally inert [16]. Polychromatic and mature erythrocytes are nucleated, non-replicating G0-phase cells. Thus, histone posttranslational modifications related to cell cycle do not confound the analyses of transcribed chromatin. Polychromatic erythrocytes express the adult β^A^-globin gene as do 15-day chicken embryo erythrocytes, but do not express the β^H^-globin gene as do cells of late embryos and newly hatched chickens [17–19]. Approximately 1–2 % of polychromatic and mature erythrocyte chromatin has dynamically acetylated histones [10, 20, 21]. Due to a particularly high density of H1/H5 linker histones [22], the bulk of chicken polychromatic erythrocyte chromatin is extremely condensed and insoluble at physiological ionic strength. However, the dynamically highly acetylated histones associated with transcriptionally active/poised chromatin prevent H1/H5 from rendering active/poised gene polynucleosomes insoluble at physiological ionic strength [9]. Exploiting these properties of chicken polychromatic erythrocyte chromatin, we designed a chromatin fractionation protocol to isolate transcriptionally active/competent chromatin. The polynucleosomes (fraction F1) are enriched in active histone marks including the dynamically highly acetylated four core histones, H3K4me3 and uH2B [23, 24]. Furthermore, F1 chromatin is enriched in u-shaped atypical nucleosomes, which were first discovered by Allfrey’s laboratory [25–27]. The nucleosomes in the F1 fraction rapidly exchange with newly synthesized histones (replication-independent class of histones) and are readily dissociated by DNA intercalators [28–30], demonstrating the lability of the F1 nucleosomes.

Our previous studies have mapped the 5′ boundary of the β-globin chromatin domain that was soluble at physiological ionic strength [11, 23]. We exploited this powerful chromatin fractionation procedure to further map the salt-soluble organization of the β-globin chromatin domain and determine whether other regions of the chicken polychromatic erythrocyte genome had domains of salt solubility akin to the β-globin chromatin domain. In conjunction with next-generation DNA and RNA sequencing (DNA-seq and RNA-seq) as well as chromatin immunoprecipitation-DNA sequencing (ChIP-seq), we could identify all the active chromosomal domains that were soluble at physiological ionic strength. Furthermore, we determined their structural signatures in relation to expression levels of genes contained within the domain. Herein, we present the functional organization of the chicken polychromatic erythrocyte genome.

## Results

### Genome-wide mapping of polychromatic erythrocyte transcribed chromosomal domains

**Fig. 1:**
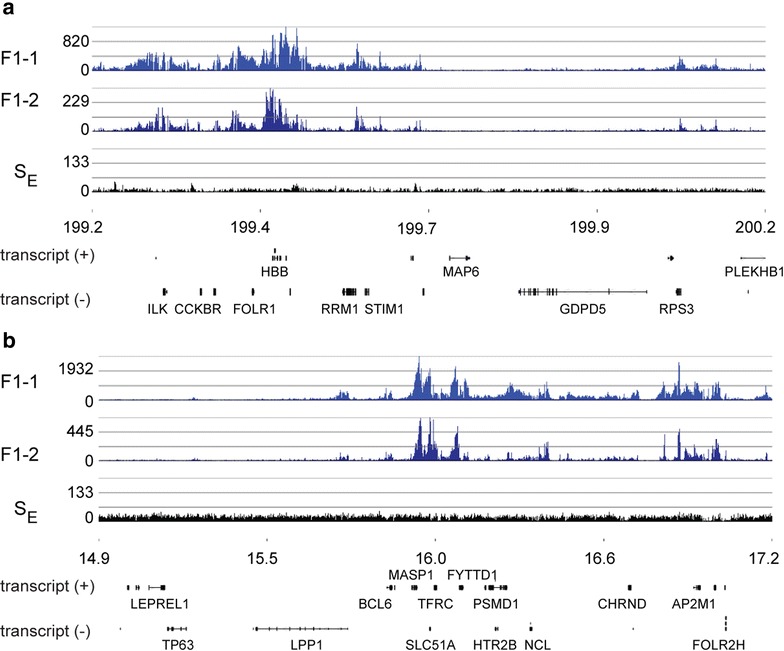
Representative browser snapshots of F1 and S_E_ chromatin DNA-seq. The DNA from two biological repeats of F1 (F1-1 and F1-2) and S_E_ chromatin fractions isolated from chicken polychromatic erythrocytes was sequenced. The positions are indicated in Mbs. **a** Region of chromosome 1. **b** Region of chromosome 9

To isolate fraction F1 chromatin, chicken polychromatic erythrocyte nuclei were incubated with micrococcal nuclease, bulk chromatin (S_E_) was released, and chromatin fragments soluble at 150 mM NaCl were isolated and size-resolved [23]. The F1 chromatin consisted of chromatin fragments ranging in size from 0.4 to 3.4 kb, with the average DNA length being 1.5 kb (Additional file 1a). Next-generation DNA sequencing of F1 chromatin generated an uneven profile with clusters of read enrichment varying in intensity and breadth, interspersed with regions depleted in reads. In contrast, the track of bulk chromatin (S_E_) was flat. These data are exemplified in Fig. 1 showing the sequence reads for a 1000-kb region on chromosome 1 and a 2300-kb region on chromosome 9. Both regions displayed long stretches (500–1000 kb) of salt-soluble chromatin interrupted with equally long stretches of salt-insoluble chromatin. Within a F1-enriched region, chromatin salt solubility was fluctuating, and when we zoomed in, we could distinguish several distinct domains within this region, for example see the β-globin (HBB) domain (Fig. 1a, Additional file 1b). The profiles generated from two biological repeats of F1 chromatin (F1-1 and F1-2) were similar. Thus, we only show tracks from F1-2 in the following figures.

**Fig. 2:**
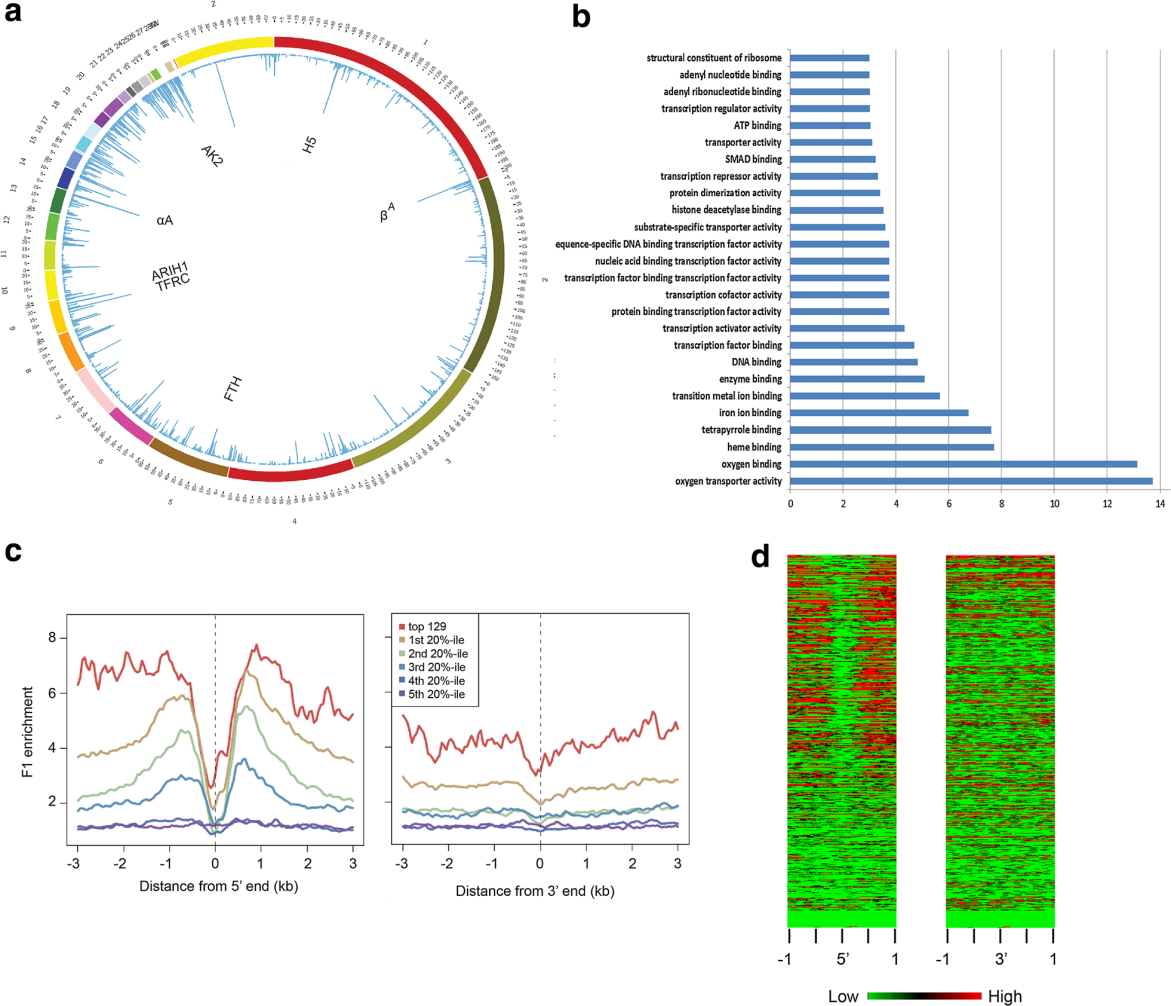
Active chromatin distribution and transcriptional activity. **a** Circos plot of DNA sequence enrichment in fraction F1 polynucleosomes. The *outer ring* represents the chicken chromosomes, and the inner ring details the peak of F1 DNA-seq reads. Some of the most enriched genes are identified. **b** Gene ontology function analysis of fraction F1. The most significantly enriched GO groups (*P* < 0.001 in one-tailed Fisher’s exact test) are displayed. The *X*-*axis* represents the −log_10_ (*P* value). **c** TSS- and TTS-centered profiles of F1 chromatin enrichment for the 129 most expressed genes and for quintile classes based on gene expression levels (Additional file 2). **d** Heatmap of F1 chromatin DNA-seq signals spanning 1 kb on each side of TSS and TTS of genes from the galGal3 RefSeq database. All 5479 genes were ranked from top to bottom, according to their level of expression (Additional file 2)

To visualize the genome-wide profiling of salt-soluble chromatin, we show a Circos plot of F1-enriched sequences (Fig. 2a). The chicken karyotype consists of 38 autosomes and a pair of sex chromosomes (ZW female, ZZ male) and is made up of macro- and microchromosomes. Several arbitrary chromosome classifications exist [31–34]. Here, we use the initial categorization, defining chromosomes 9-38 and W as cytologically indistinguishable microchromosomes [35]. Early studies estimated that microchromosomes constitute 23 % of the chicken genome and contain 48 % of all genes [32]. In agreement, sequencing of the genome showed that gene density is inversely correlated with chromosome length [33]. As seen in the Circos plot of F1-enriched sequences (Fig. 2a), there was a higher density of salt-soluble chromatin in polychromatic erythrocytes on microchromosomes than on macrochromosomes.

The F1 reads were used to rank genes contained within salt-soluble chromatin domain. The rank order of these genes was used for GO term analysis. In terms of molecular functions, genes involved in the hemoglobin synthesis pathway were the predominant sets of active genes in F1 chromatin, followed by genes encoding proteins involved in transcription regulation (Fig. 2b). In summary, the chicken polychromatic erythrocyte genome is organized in clusters of discrete salt-soluble chromatin domains, and these expanses of chromatin exhibiting an open structure alternate with long stretches of salt-insoluble chromatin.

### Chromatin domain organization correlates with gene expression levels

Snapshots of F1 and S_E_ chromatin DNA-seq confirmed that the active β^A^-globin resided in a salt-soluble domain (HBB), while sequences from the inactive ovalbumin locus are depleted in F1 (Fig. 1 and Additional file 1b). To determine the correlation between transcriptional activity and chromatin salt solubility at the genome-wide level, the chicken polychromatic erythrocyte transcriptome was characterized by cellular RNA-seq analyses. The 5479 galGal3 RefSeq genes annotated in the UCSC genome database were placed in order of their level of expression (Additional file 2). RNA-seq assessment of cellular transcript levels by RPKM was validated by RT-qPCR analyses. We show that genes with high (*HBG2*), intermediate (*CA2* and *FTH1*) or low (*HDAC2* and *PRMT7*) RPKM values had relatively similar transcript levels in our validation studies (Additional file 3). We also isolated and sequenced nuclear RNA and found a very high correlation (*r* = 0.82) between cellular and nuclear RNA-seq data sets, as seen in the scatterplot and snapshots of β-globin and *H1F0* (coding for H5) transcripts (Additional file 4). Moreover, RNA-seq data analysis revealed that for the most highly expressed genes, that is about the first 20th-percentile class, there was a low level (about 1 %) of antisense transcription of the coding region (Additional file 5). This antisense transcription was observed for coding regions of cellular and nuclear transcripts (Additional file 4).

There are two types of histone genes; those that are replication dependent and those that are replication independent. The polychromatic erythrocyte, which has ceased replication, had low expression of replication-dependent histone H1 (*HIST1H1C*), H2A (*HIST2H2AC_dup2*), H2B (*HIST1H2BO*, *H2B*-*V*) and H4 (*H4*, *H4*-*VII*). However, expression of replication-independent histone genes (*H3F3C*, *H2AFZ*, *H1F0*) was high.

Chicken erythroid progenitor cells undergo a restructuring of the cytoskeleton during the terminal differentiation program [36]. We observed that the polychromatic erythrocytes expressed several cytoskeleton-associated genes such as *SPTAN1* (spectrin, alpha, non-erythrocytic 1) gene, *EPB41* (protein 4.1), genes (*ANKHD1*, *ANKRD27*) coding for ankyrin repeat domain proteins, and spectrin genes (*SPTAN1*, spectrin, alpha, non-erythrocytic 1 and *SPTBN1*, spectrin, beta, non-erythrocytic 1). However, the polychromatic erythrocytes did not express ankyrin genes (*ANK1*, *ANK2*, *ANK3*), erythrocytic-specific spectrin genes *SPTA1* (spectrin, alpha, erythrocytic 1), *SPTB* (spectrin, beta, erythrocytic), or band 3 gene/anion exchange gene 1 (*SLC4A1/AE1*).

To determine whether enrichment in F1 chromatin paralleled gene expression levels, the 5479 genes placed in order of their level of expression (Additional file 2) were divided into five 20th-percentile classes in relation to expression level. For each class, as well as for the top 129 expressors (number chosen to include *H1F0* gene known to be expressed in polychromatic erythrocytes [17]) , sequence enrichment in F1 chromatin was analyzed at the transcription start site (TSS) and termination site (TTS) of each gene (Fig. 2c). The first 20th-percentile group with highest gene expression levels, and even more so the top 129 expressors, showed the highest sequence enrichment in F1 chromatin, while the last two 20th-percentile groups were not enriched, further validating the ability of this salt fractionation method to isolate transcriptionally active chromatin. For all classes, enrichment in F1 chromatin was higher at the TSS than at the TTS, although the difference between F1 enrichments at TSS and TTS was not as marked for the top 129 expressors. The sequence enrichment profile extending over 3 kb on both sides of nucleosome-free TSS demonstrated that solubility of chromatin at physiological ionic strength was not limited to the first nucleosome of the gene as in the case of other chromatin sources (Fig. 2b) [37, 38]. Heatmaps of F1 chromatin DNA-seq reads around the TSS and TTS of the 5479 genes ranked from top to bottom were consistent with the enrichment plots for the quintile classes, showing a marked enrichment for about the top 60 % of expressors around the TSS, but for only about 10 % around the TTS (Fig. 2d).

Regarding the chromosomal location, microchromosomes held 43 % of the genes from the first 20th-percentile group. Slightly more of the actively expressed genes (56 %) in the first 20th-percentile group were located on the macrochromosomes. Thus, the genomic distribution of the most active genes in polychromatic erythrocytes was slightly in favor of the macrochromosomes. To conclude, there was an overall correlation between levels of gene expression and the extent of salt solubility of their associated chromatin.

### Features of salt-soluble chromatin

To compliment the F1 chromatin sequence and transcriptome analyses, we mapped the positions of two active chromatin marks (H3K4me3 and H3K27ac) (Additional file 6). H3K27ac is the signature of active enhancers and promoters [39], while H3K4me3 maps to the 5′ end of the body of active genes in mammals [19, 40, 41]. H3K27ac or H3K4me3 average coverage around the TSS was determined for each of the 20th-percentile classes described above. Both H3K27ac and H3K4me3 were only significantly enriched in the 5′ region of the most highly expressed genes (first 20th-percentile). The average profile was sharper for H3K4me3 than H3K27ac, with H3K4me3 peaking between 0.5 and 1.5 kb downstream of the TSS. Consistent with these data, H3K4me3 and H3K27ac heatmaps spanning 1 kb on each side of the TSS showed enrichment for the top 40 % expressors (Additional file 6).

**Fig. 3:**
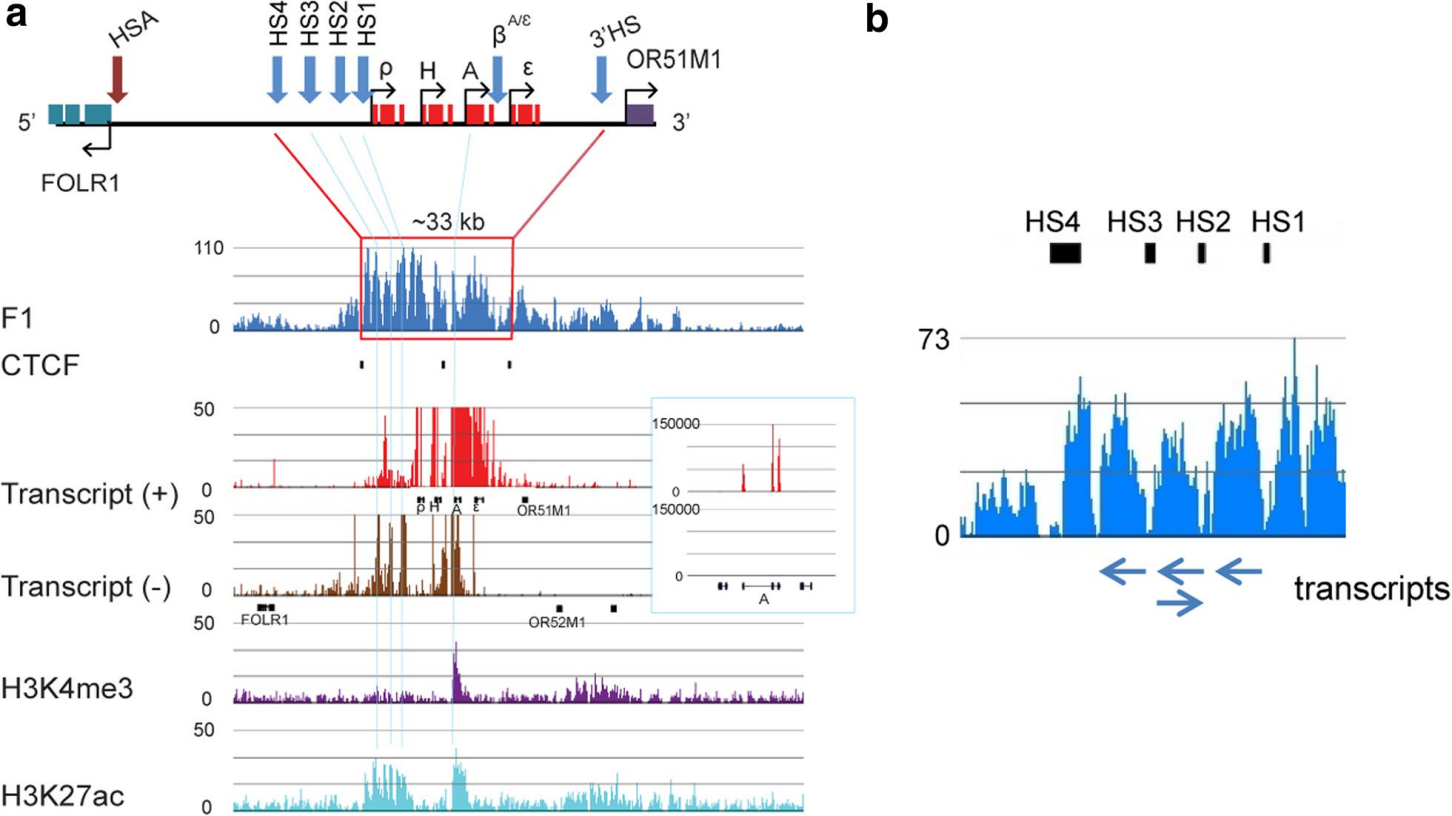
Chromatin profile and transcriptional activity of β-globin domain. **a** Schematic of the β-globin domain, detailing the developmentally regulated β-globin genes and DNAse I-hypersensitive sites (HS4 and 3′HS delimitating the locus). HS1, HS2, HS3 and β^A/ε^ enhancer are collectively known as locus control region (LCR) and regulate the expression of the four β-globin genes. Below the maps, are signal tracks showing DNA enrichment in F1 fraction, CTCF-binding sites (as *vertical bars*), transcripts on (+) and (−) strands and H3 modifications. mRNAs (with exons as *black boxes*) are shown below their template strand. The *inset* to the right shows the level of transcripts on an expanded scale. *Vertical blue lines* illustrate the position within the domain of prominent features (H3K27ac and/or H3K4me3 peaks and eRNAs). **b** Amplification of signal tracks showing F1-enriched DNA and transcribed RNAs in the β-globin LCR region

Genes from the first 20th-percentile group had distinct salt-soluble chromatin organizations. The genes with the highest expression were present in broad salt-soluble chromatin regions, while moderately or poorly expressed genes had the salt-soluble chromatin confined to their 5′ regions. To illustrate the broad salt-soluble domains, we show the chromatin profile of the β-globin locus. Figure 3a shows that salt solubility co-mapped with the well-known 33-kb β-globin domain, as defined by DNase I sensitivity, histone acetylation and CTCF-binding sites marking the boundaries. Moreover, within the domain, F1 enrichment reads paralleled the high acetylation profile [6, 42, 43]. Similar data were obtained for the α-globin locus [4] (data not shown). Beside the abundant β^A^-globin mRNA and low level of antisense transcription (about 1 % of sense transcript), we detected LCR-associated RNAs or enhancer-derived RNAs (eRNAs), which originated from the HS1, HS2 and HS3 sites (Fig. 3a, b). Attribution of transcriptional activity from β^A/ε^ enhancer was precluded by the massive β^A^ gene transcription. The H3K27ac mark was positioned at HS1, HS2, HS3 and β^A/ε^ enhancer, as well at the promoter and along the body of the β^A^ gene, while H3K4me3 was enriched in the body of the β^A^-globin gene (Fig. 3a). These results demonstrate that the β-globin genes are present in a salt-soluble chromatin domain, with the boundaries of the 33-kb domain defined by a loss of a salt-soluble chromatin structure. The LCR chromatin region is organized into salt-soluble chromatin regions enriched in H3K27ac, with MNase-hypersensitive sites demarcating the boundaries of each region of the LCR.

We looked in detail at the chromatin features of nine other genes among the 129 top expressors and found out that those genes resided in broad salt-soluble chromatin domains: the α-globin *(HBA)* gene (expressor # 1, in a 60-kb domain), *CA2* (expressor # 13, in a 86-kb domain), *FTH1* (expressor # 14, in a 46-kb domain), IFRD1 (expressor # 21, in a 33-kb domain), NCOA4 (expressor # 23, in a 22-kb domain), *TFRC* (expressor # 51, in a 35-kb domain), *ARIH1* (expressor # 125, in a 154-kb domain), *AK2* (not annotated in the galGal3 RefSeq gene database, in a 44-kb domain) and *H1F0* (expressor # 129, in a 48-kb domain).

**Fig. 4:**
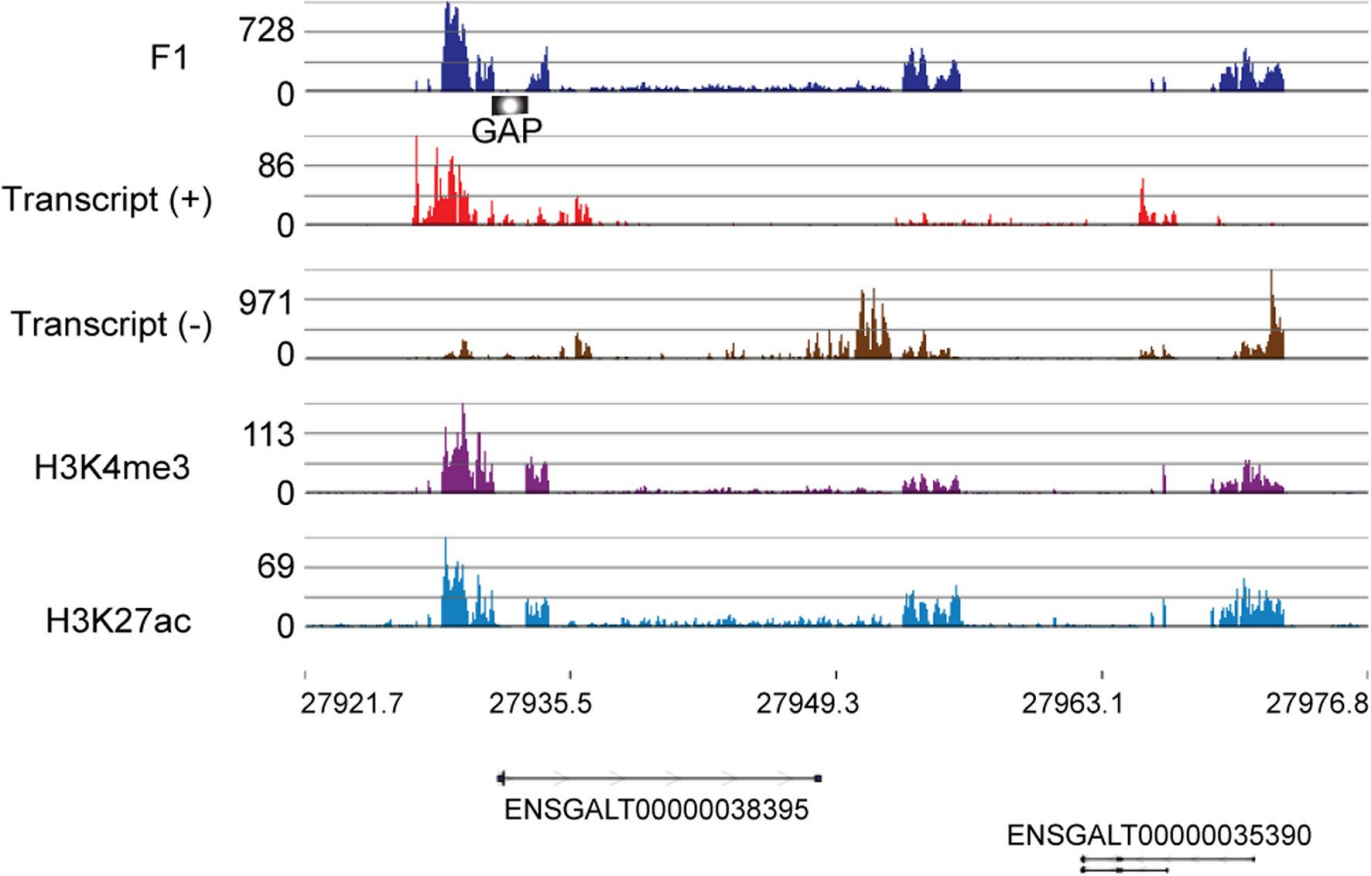
Chromatin profile and transcriptional activity of region of interest on chromosome Z. The positions are indicated in kbs. It should be noted that the dips in the F1-enrichment, H3K4me3 and H3K27ac profiles are due to a gap in the genome sequence

As to genes associated with a salt-soluble chromatin limited to their 5′ regions, the Circos plot (Fig. 2a) displayed a very high F1 enrichment of chromatin (at approximately 28,000,000) on the sex chromosome Z. This peak was mapped to a region containing two MHM (male hypermethylated) locus genes believed to play a role in localized dosage compensation (Fig. 4). The two genes *ENSGALG00000023324* (transcript: ENSGALT00000038395) and *ENSGALG00000018479* (transcript: ENSGALT00000035390) showed a large increase in expression in gonads of female (ZW) chickens compared to male (ZZ) chickens [44, 45]. They code for uncharacterized proteins of 103 and 60 amino acids, respectively. The *ENSGALG00000018479* gene was found overexpressed in the brain (hypothalamus and thalamus) of 21-day-old females compared to males [44]. Our results show that the salt-soluble F1 chromatin on the chromosome Z identified the presence of the MHM locus genes (Fig. 4).

**Fig. 5:**
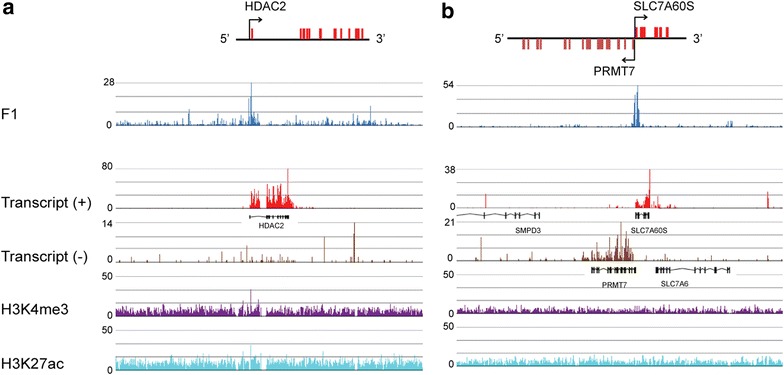
Chromatin profile and transcriptional activity of moderately and poorly expressed genes. **a** *HDAC2*. **b** *PRMT7*

Other genes with very small region of salt-soluble chromatin at their 5′ end or body were moderately or poorly expressed in chicken polychromatic erythrocytes, e.g., *HDAC2* (histone deacetylase 2) and *PRMT7* (protein arginine methyltransferase 7) (Fig. 5). No particular feature (H3K27ac or H3K4me3) or enhancer-associated chromatin feature could be identified for either gene.

Our results have identified several domains that have extended salt-soluble chromatin domains similar to the α- and β-globin gene domains. The genes with this chromatin organization tend to be highly expressed. A larger number of genes, which are expressed at lower levels, have a salt-soluble chromatin organization confined to the 5′ end of the gene.

## Discussion

Our results demonstrate that the broad dynamically acetylated, salt-soluble chromatin domain organization of the α- and β-globin genes is a characteristic of many highly expressed genes in the chicken polychromatic erythrocytes. The boundaries of the salt-soluble chromatin containing the α- and β-globin genes mapped precisely with the boundaries defined by highly acetylated histones (H3K9/14ac; acetylated H4). For highly expressed genes, the broad salt-soluble, dynamically acetylated regions were present 5′ and 3′ to the TSS and sustained to lesser extent around the TTS. It is possible that the antisense transcripts are a feature of the highly acetylated chromatin state of these genes. It is of note that antisense transcripts for the α- and β^A^-globin genes have been reported previously [7, 46]. Less actively expressed genes have highly acetylated F1 chromatin regions restricted to their 5′ ends. This restricted highly acetylated domain is typical of mammalian genes [3].

The majority of the polychromatic erythroid chromatin is highly condensed due to the excessive amount of histone H5 and the low acetylated state of the bulk of chromatin. Nevertheless, we find that the genomic distribution of the H3K4me3 at the 5′ of the coding region of expressed genes and the presence of H3K27ac at enhancers and LCR are typical of mammalian cells. It is also noteworthy that the repressive environment in chicken polychromatic erythrocytes also facilitated its transcriptome characterization. Typically, high-throughput sequencing of steady-state cellular RNA is not a suitable method to detect the rarer and/or less stable transcripts, resulting from antisense transcription or originating from enhancers [47, 48]. However, cellular RNA-seq analyses allowed us to identify such transcripts. For the β^A^-globin LCR, we observed transcripts originating from HS1, HS2 and HS3 sites. This is in contrast to human erythroid cells in which RNA polymerase II-mediated transcription from one of the LCR elements goes in the globin mRNA sense direction [49, 50]. The transcription in chicken polychromatic erythrocytes occurred on the (−) strand from HS2 and HS1 and in both directions from HS3. In contrast to the other LCR-hypersensitive sites (HS2, HS3 and β^A/ε^), HS1 does not have independent enhancing activity [51], but is likely to play a role in transcription regulation as it presents the traits of an active enhancer.

Studies on the organization of chicken chromosomes show that microchromosomes are gathered within the nuclear interior, while macrochromosomes are located at the periphery of nuclei in both cycling fibroblast and non-proliferating neurons, suggesting that this radial arrangement may exist in erythrocytes [32]. On the other hand, Hutchison and Weintraub reported that the DNase I-sensitive chromatin was located on the periphery of chromosomal territories, along interchromatin channels in chicken erythrocytes [52]. Regardless of gene chromosomal location, transcriptionally active/poised chromatin domains likely share a similar compartmentalization, looping out of their chromosome territories [52, 53]. The solubility and location of the transcriptionally active chromosomal domains in the nuclear environment ensures their ready access by transcription factors and chromatin modifying and remodeling factors.

Chicken has long been recognized as a model system to study the organization and function of a vertebrate genome [54]. Its genome is almost three times smaller than the human genome, but has about the same number of genes, with 60 % of them having a single human ortholog. Moreover, there are long blocks of conserved synteny between the chicken and human genomes [33]. In terms of chromosomal organization of genes, the human genome is closer to the chicken than to rodents. Additionally, following 310 million years of separate evolution, conserved noncoding sequences are likely to highlight functional elements in both chicken and human genomes [33]. Thus, our studies supply valuable information on the structural and functional organization of the chicken polychromatic erythrocyte epigenome and may also provide insights into the human erythrocyte genome organization.

## Conclusions

One to two percent of the chicken polychromatic erythrocyte epigenome is associated with dynamically acetylated histones. As the salt solubility of chromatin containing expressed genes depends on the state of the dynamically acetylated histones, we would expect that a similar percentage of the polychromatic erythrocyte chromatin would be soluble at physiological ionic strength. We show that expressed genes are organized in broad dynamically highly acetylated, salt-soluble chromatin domains containing at least one highly expressed gene or in narrow dynamically acetylated, salt-soluble chromatin regions restricted to the 5′ end of moderately or poorly expressed genes. The bulk of the genome is highly compacted and silent. The genomic mapping of salt-soluble chromatin domains will aid in the annotation of genes expressed in erythroid cells.

## Methods

### Isolation of chicken erythrocytes

Polychromatic erythrocytes were isolated from anemic female adult white Leghorn chickens as described [23]. Ethical approval was obtained from the University of Manitoba Animal Care Committee. All methods involving the use of chicken were approved by the committee and carried out in accordance with its guidelines and regulations. The birds were purchased through Central Animal Care Services, University of Manitoba and were housed under standard conditions. A biological sample consisted of a pool of red blood cells from 11 to 12 anemic chickens (see Additional file 7 for age and weight ranges).

### Salt fractionation

Chicken polychromatic erythrocyte nuclei were prepared as described [23, 28]. The equivalent of 50 A_260_ nuclei were incubated with 1.5 unit of micrococcal nuclease (Worthington Biochemical Corporation) for 12 min at 37 °C, and the digestion was stopped by the addition of EGTA to 10 mM. Chromatin fragments soluble in a low ionic strength solution containing 10 mM EDTA were recovered in fraction S_E_. Chromatin fraction S_E_ was made 150 mM in NaCl, and chromatin fragments from the salt-soluble fraction (S_150_) were size-resolved on a Bio-Gel A-1.5 m column to isolate the F1 fraction containing polynucleosomes [24].

### ChIP-seq assays

ChIP-seq assays, using antibodies against H3K27ac or H3K4me3 from Abcam, were done as previously described [24, 55], except that chicken polychromatic erythrocyte nuclei were treated with 0.5 % formaldehyde and chromatin was sheared into 200-bp fragments. See Additional file 8 for details regarding sequencing data.

### Sequencing and mapping of data

DNA libraries and strand-specific (100–250 nucleotides) RNA libraries (prepared with the SOLiD Total RNA-Seq kit) were sequenced on the 5500 × l SOLiD™ System [55]. Single-end sequence reads of 50 bp in length were generated from the S_E_ control sample and two biological replicates of F1 (F1-1 and F1-2) chromatin. In total, 70–80 % of these color-space sequence reads were mapped to the chicken reference genome galGal3 using the LifeScope™ Genomic Analysis Software 2.5.1 (Life Technologies). Mismatch penalty of -2 and a minimum mapping quality score of 8 were applied in mapping parameter settings. See Additional file 8 for details about F1 and S_E_ tracks.

Two biological replicates of cellular RNA-seq generated a total of 120 million paired-end (50 × 35 bp) sequence reads, and more than 85 % of these reads were mapped to the genome. 110 million paired-end reads were generated from two nuclear RNA-seq samples. More than 85 % of these paired-end reads were mapped to the genome. The sense and antisense RNA track data were extracted from BAM files using SAMtools [56]. See Additional file 8 for details about Transcript (+) and Transcript (−) tracks.

H3K27ac and H3K4me3 ChIP-seq produced approximately 30 and 24 million sequence reads, respectively, and more than 65 % of these sequences were mapped to galGal3 with an average mapping quality value of 63. We also generated 32 million sequence reads from the input sample.

The mapped BAM or WIG files were visualized using tools from the Integrative Genome Viewer (IGV), UCSC Genome Browser, or Partek Genomic Suite v6.6. The genes were annotated using Ensembl transcripts database release-70 or UCSC RefSeq genes.

### RNA isolation and real-time RT-qPCR analysis

Total RNA was isolated from polychromatic erythrocyte cells, and nuclear RNA was isolated from nuclei using the RNeasy Mini kit (QIAGEN) according to manufacturer’s instructions. Complementary DNA was generated from total RNA (800 ng) using the iScript cDNA Synthesis kit (BioRad) following the manufacturer’s specifications. SsoAdvanced universal SYBR^®^ Green supermix (BioRad) was used to perform real-time PCRs using 5 ng of cDNA on a CFX96 Touch™ Real-Time PCR Detection System (BioRad). The primers used for RT-PCR are listed in the Additional file 9. The RNA levels were normalized against 18S rRNA.

### Active chromatin detection and genomic distribution

We applied a clustering approach (SICER) [57] for identification of islands of DNA-seq enrichment using F1 DNA-seq-mapped BAM files as inputs. The window and gap sizes were chosen to be 1 kb each. The S_E_ DNA-seq data were used for background subtraction. We found a total of 9466 islands with FDR < 0.1. The island scores were transformed to *z*-scores = (*x* − *m*)/*σ* where (*x*) is the island score, (*m*) is the mean of all island scores and (*σ*) is the standard deviation of all island scores. The z-scores were plotted to the galGal3 genome using Circos [58].

### Chromatin profiling of transcriptionally active genes

Transcriptional levels were detected using the LifeScope whole-transcriptome mapping module. We used the reads per kilobase per million (RPKM) to assign gene transcription levels. The cellular RNA-seq duplicates were averaged for each gene, and these values were used to classify galGal3 RefSeq genes into five 20 percentile groups. The cis-regulatory element annotation system (CEAS) [59] was used to profile these five gene lists against the F1 DNA-seq data. The profiles for regions spanning 1 kb on each side of TSS and TTS were plotted. The F1 DNA-seq data extracted at TSS and TTS regions (−1 to 1 K) of ranked genes were displayed per 10-base bin on heatmaps by a R script.

## Data availability

The sequencing data are available from GEO under accession number GSE75955.

## Additional files

10.1186/s13072-016-0068-2 **a** Fractionation of avian erythrocyte chromatin. Chicken polychromatic erythrocyte nuclei were incubated with micrococcal nuclease, and chromatin fragments soluble in a low ionic strength solution containing 10 mM EDTA were recovered in fraction S_E_. Chromatin fraction S_E_ was made 150 mM in NaCl, and chromatin fragments from the salt-soluble fraction (S_150_) were size-resolved on a Bio-Gel A-1.5 m column to isolate the F1 fraction containing polynucleosomes. **b** β-globin and ovalbumin F1 and S_E_ chromatin profiles. The DNA from F1 and S_E_ chromatin fractions isolated from chicken polychromatic erythrocytes was sequenced. The signal tracks show DNA enrichment for β-globin on chromosome 1 and OVAL (ovalbumin) on chromosome 2.
10.1186/s13072-016-0068-2 Gene ranking according to expression levels in chicken polychromatic erythrocytes. All 5479 genes from the galGal3 RefSeq database were placed in order of their level of expression (mean RPKM from two biological samples) and were then divided into five 20th-percentile classes in relation to expression level. First 20 %-ile (*red filled*), second 20 %-ile (*green filled*), third 20 %-ile (*blue filled*), fourth 20 %-ile (*dark blue filled*) and fifth 20 %-ile (*violet filled*).
10.1186/s13072-016-0068-2 Validation of RNA-seq data by RT-qPCR. Comparison of RPKM values from RNA-seq analyses with RNA levels determined by RT-qPCR assays for specific genes. Transcript levels were normalized to 18S rRNA levels. * Three RT-qPCR assays were done on three different RNA preparations from Sample 3.
10.1186/s13072-016-0068-2 Transcriptional activity determination by cellular and nuclear RNA-seq. **a** Signal tracks showing DNA enrichment in F1 fraction for β-globin (HBB) locus and transcripts on (+) and (−) strands from cellular and nuclear RNA are shown. mRNAs (with exons as black boxes) are shown below their template strand. **b** Same for H5. **c** Correlation of the cellular and nuclear RNA-seq data. Unit on both axes is log_2_ RPKM, with RPKM being the average of two biological repeats.
10.1186/s13072-016-0068-2 Sense and antisense transcript reads for the 1095 genes from the galGal3 RefSeq database belonging to the first 20th-percentile. 
10.1186/s13072-016-0068-2 H3K4me3 and H3K27ac profiles as a function of gene expression. TSS-centered profiles were divided into quintile classes based on gene expression levels (Additional file 2). Below, heatmap spanning 1 kb on each side of TSS and TTS of all genes ranked from top to bottom, according to their expression levels (Additional file 2).
10.1186/s13072-016-0068-2 Description of polychromatic erythrocyte sample sources. Each sample consisted of red blood cells collected from 11 to 12 anemic chickens: age and weight ranges.
10.1186/s13072-016-0068-2 Table with information regarding all sequencing tracks shown in this study (total read counts, mapped read counts, MAPQ threshold and median, percentage of reads uniquely mapped).
10.1186/s13072-016-0068-2 Primers for RT-qPCR analyses.

## Abbreviations

ChIP: chromatin immunoprecipitation
eRNA: enhancer-derived RNA
F1 chromatin: salt-soluble fraction containing polynucleosomes
H3K4me3: histone H3 trimethylated at lysine 4
H3K27ac: histone H3 acetylated at lysine 27
seq: sequencing
