# Supplementary figures and images for: The chicken erythrocyte epigenome
Source: Epigenetics Chromatin. 2016 May 24;9:19. doi: 10.1186/s13072-016-0068-2 (PMC4879735; doi:10.1186/s13072-016-0068-2)

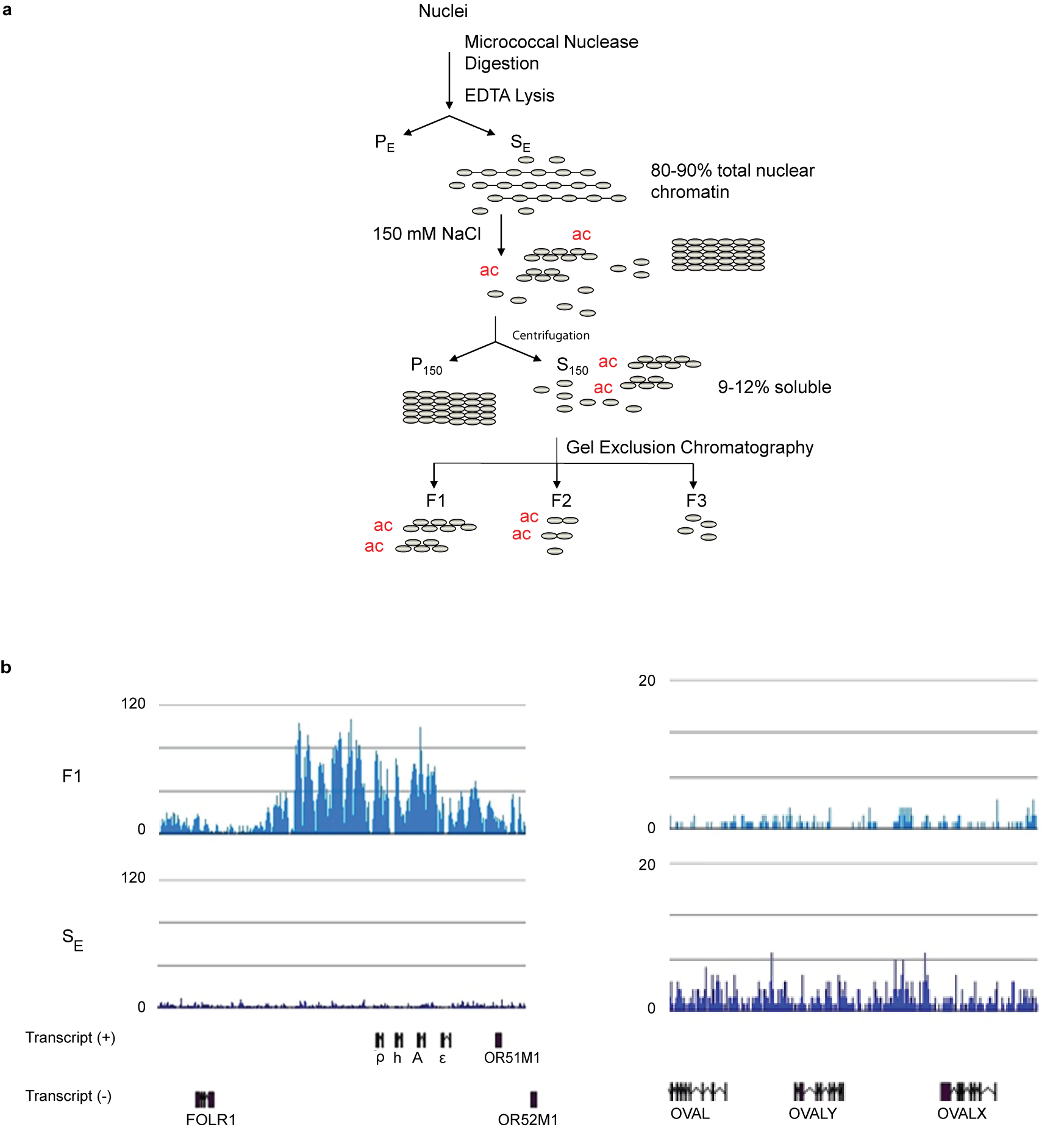

Supplement: Supplementary file 1 — 10.1186/s13072-016-0068-2 a Fractionation of avian erythrocyte chromatin. Chicken polychromatic erythrocyte nuclei were incubated with micrococcal nuclease, and chromatin fragments soluble in a low ionic strength solution containing 10 mM EDTA were recovered in fraction SE. Chromatin fraction SE was made 150 mM in NaCl, and chromatin fragments from the salt-soluble fraction (S150) were size-resolved on a Bio-Gel A-1.5 m column to isolate the F1 fraction containing polynucleosomes. b β-globin and ovalbumin F1 and SE chromatin profiles. The DNA from F1 and SE chromatin fractions isolated from chicken polychromatic erythrocytes was sequenced. The signal tracks show DNA enrichment for β-globin on chromosome 1 and OVAL (ovalbumin) on chromosome 2. [file 13072_2016_68_MOESM1_ESM.png]

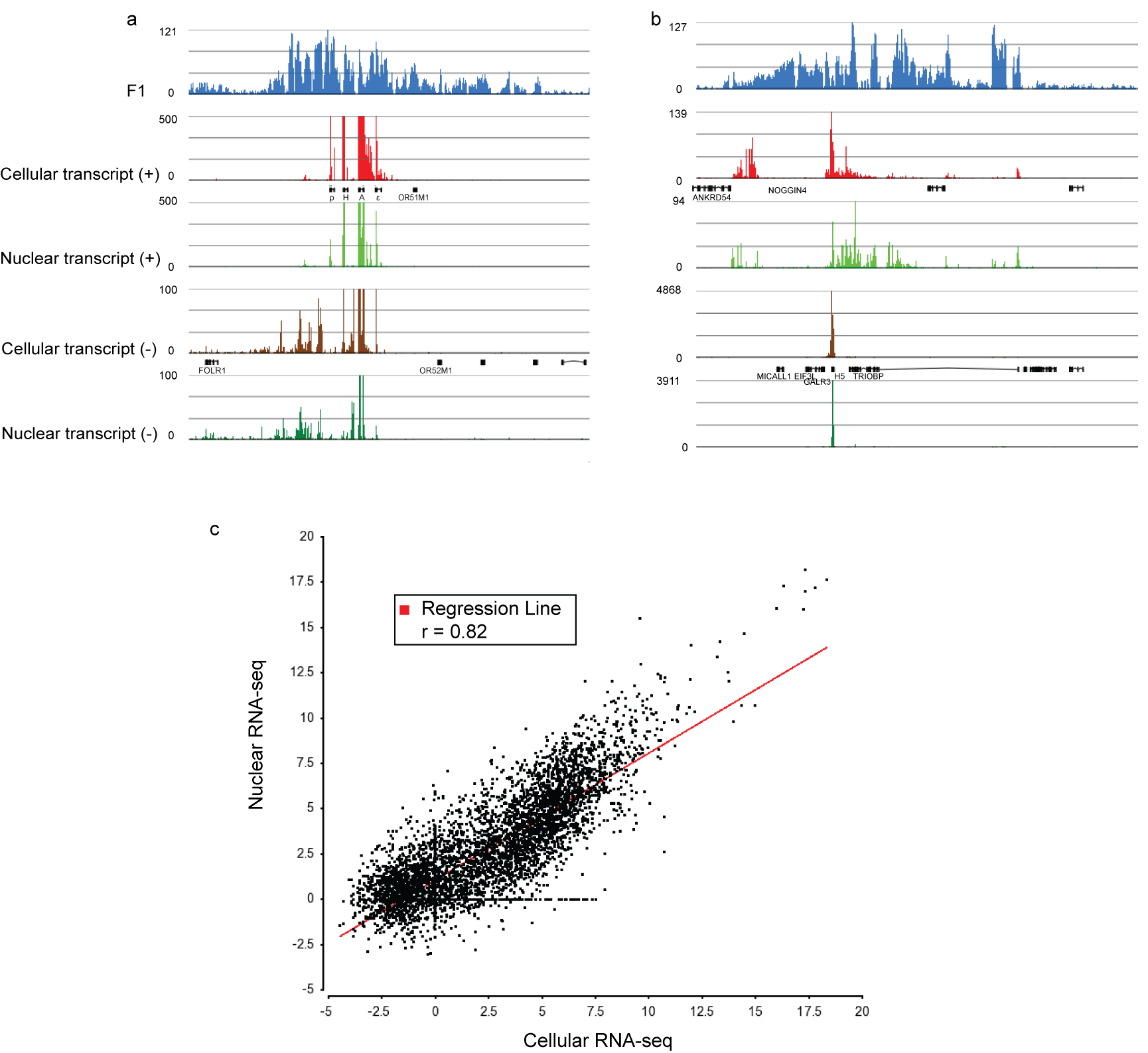

Supplement: Supplementary file 4 — 10.1186/s13072-016-0068-2 Transcriptional activity determination by cellular and nuclear RNA-seq. a Signal tracks showing DNA enrichment in F1 fraction for β-globin (HBB) locus and transcripts on (+) and (−) strands from cellular and nuclear RNA are shown. mRNAs (with exons as black boxes) are shown below their template strand. b Same for H5. c Correlation of the cellular and nuclear RNA-seq data. Unit on both axes is log2 RPKM, with RPKM being the average of two biological repeats. [file 13072_2016_68_MOESM4_ESM.png]

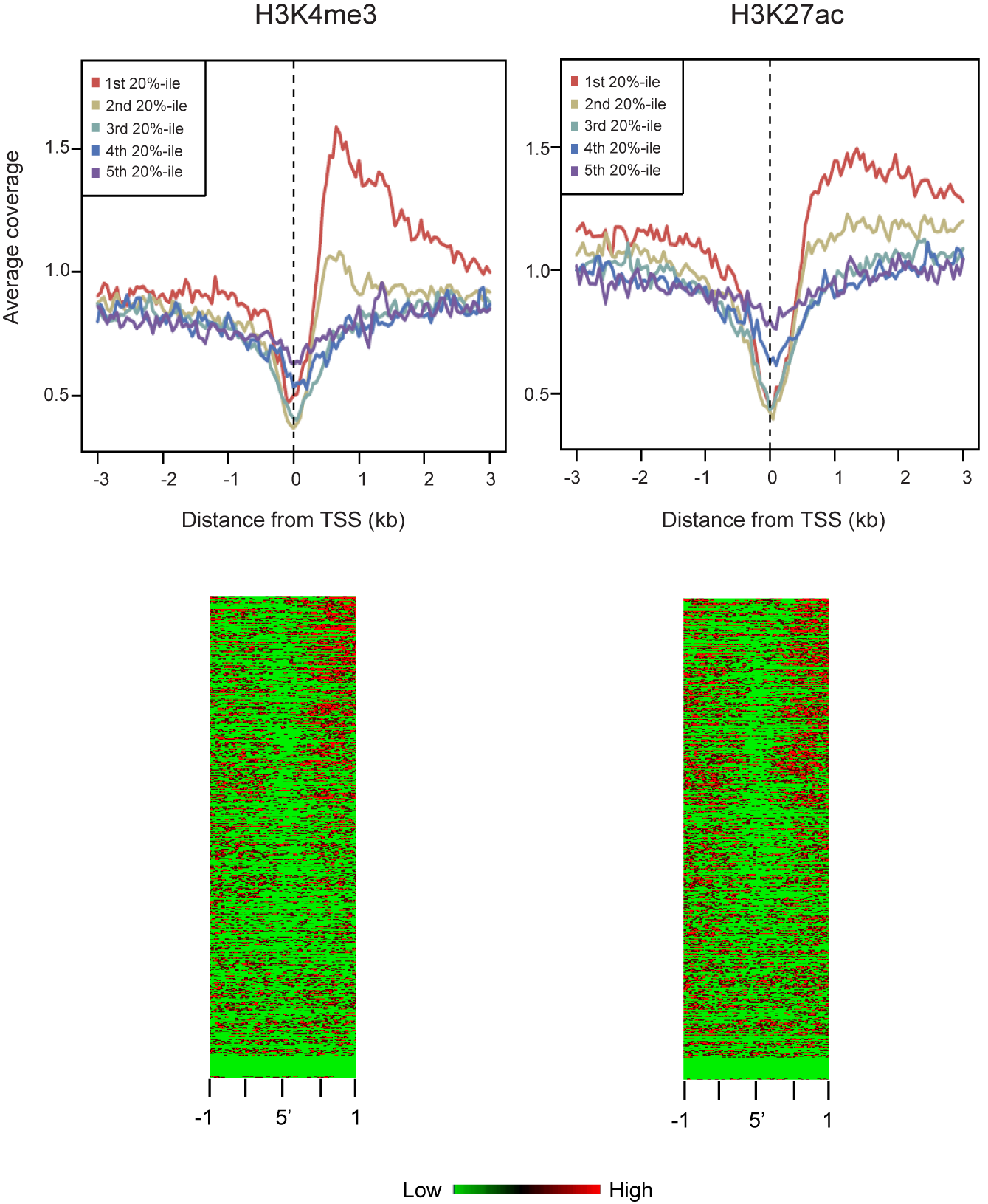

Supplement: Supplementary file 6 — 10.1186/s13072-016-0068-2 H3K4me3 and H3K27ac profiles as a function of gene expression. TSS-centered profiles were divided into quintile classes based on gene expression levels (Additional file 2). Below, heatmap spanning 1 kb on each side of TSS and TTS of all genes ranked from top to bottom, according to their expression levels (Additional file 2). [file 13072_2016_68_MOESM6_ESM.png]
