# Supplementary material for: The chicken erythrocyte epigenome
Source: Epigenetics Chromatin. 2016 May 24;9:19. doi: 10.1186/s13072-016-0068-2 (PMC4879735; doi:10.1186/s13072-016-0068-2)
Supplement: Supplementary file 9 — 10.1186/s13072-016-0068-2 Primers for RT-qPCR analyses. [file 13072_2016_68_MOESM9_ESM.docx]

**Primers for RT-qPCR assays**

| Primers | Sequences |
| --- | --- |
| HBG2-F  HBG2-R | 5'-GGCAAGAAAGTGCTCACCTC-3'  5'-GCTTGTCACAATGCAGTTCG-3' |
| FTH1-F  FTH1-R | 5'-ATTTTGACCGGGATGATGTG-3'  5'-TGGTTTTGCAGCTTCATCAG-3' |
| CA2-F  CA2-R | 5'-AGCCCCTCAGCTTCAGCTAC-3'  5'-ACTTGTCGGAGGAGTCGTCA-3' |
| HDAC2-F  HDAC2-R | 5'-TATGGACAAGGGCATCCAAT-3'  5'-CACGTAAATTTCCATTTTCCTGT-3' |
| PRMT7-F  PRMT7-R | 5'-TTCTCAACCCAAATCCATCC-3'  5'-GCGTGGTTTGCTGAGAGC-3' |
| 18S-F  18S-R | 5'-GTAACCCGTTGAACCCCATT-3'  5'-CCATCCAATCGGTAGTAGCG-3' |
